# Spotlight on PSMA as a new theranostic biomarker for bladder cancer

**DOI:** 10.1038/s41598-021-89160-0

**Published:** 2021-05-07

**Authors:** Maria Maddalena Tumedei, Sara Ravaioli, Federica Matteucci, Monica Celli, Ugo De Giorgi, Roberta Gunelli, Maurizio Puccetti, Giovanni Paganelli, Sara Bravaccini

**Affiliations:** 1 IRCCS Istituto Romagnolo per lo Studio dei Tumori (IRST) “Dino Amadori”, Via P. Maroncelli 40, 47014 Meldola, Italy; 2 Department of Urology, Morgagni Pierantoni Hospital, Forli, Italy; 3 Azienda Unità Sanitaria Locale (AUSL) Imola, Imola, Italy

**Keywords:** Cancer, Cell biology, Oncology, Urology

## Abstract

Bladder cancer (BCa) patients are diagnosed by cytology and cystoscopy. However, these diagnostic tests bear some limitations. We sought for reliable biomarkers to better determine BCa extension. Prostate-specific membrane antigen (PSMA) appears to fulfill this requirement in prostate cancer but its role in BCa has not been established yet. We then analyzed 87 bladder tissue samples from 74 patients assessing PSMA expression by immunohistochemistry. The median PSMA expression, exclusively found in tumor neovasculature, in terms of H-score significantly differed between non-tumor samples and tumor samples (*p* = 0.00288) showing a higher neovasculature-related PSMA expression. No differences were observed in relation to tumor type, grade and stage. BCa neovasculature-related PSMA overexpression may be useful in defining the degree of extension of the neoplasm. In addition, testing PSMA expression by immunohistochemistry may hold theranostic implications both considering anti-angiogenesis agents and radio-labelled PSMA ligands for intracavitary radionuclide therapy. In our opinion, BCa neovasculature-related PSMA overexpression may be considered an apt target for anti-angiogenesis and radionuclide treatment in BCa, once the evaluation of tumor-retention time for the appropriateness of long half-life therapeutic PSMA ligands as radionuclide treatment will be performed.

## Introduction

Bladder cancer (BCa) is the 10th most common form of cancer worldwide, with 549,000 new cases estimated and 200,000 deaths^1^. Despite urothelial BCa has been reported to be 3 times more common in men, many studies conclude that women have poorer prognosis^2^.

The excess risk in men has been hypothesized to be driven by higher exposure to carcinogens (cigarette smoking, occupation, chemical and water contaminants), whereas the reasons for poorer survival in women is not clear^2^.

The most common type of BCa is urothelial carcinoma, and approximately 75% of cases are non–muscle invasive (NMIBC) at diagnosis. Generally, NMIBC has a favorable prognosis and is diagnosed and treated with trans-urethral endoscopic resection, followed by intravesical therapy if the risk of recurrence of NMIBC or progression to muscle-invasive bladder cancer (MIBC) is high^3^. Cytology and cystoscopy are the conventionally used techniques for BCa diagnosis and monitoring but these have some limitations. The former has low sensitivity for low grade and low stage tumors, the latter has high specificity but it is invasive, therefore new diagnostic biomarkers would be desirable.

MIBC is found in about 25% of patients at the time of diagnosis, and these are conventionally treated by means of radical cystectomy (RC) and lymph node dissection^4^. The presence of nodal metastases, found in 20% to 25% of patients at the time of RC, is an important indicator for poor outcome along with pathologic tumor stage^5^. However, up to 37% of patients with locally-advanced BCa and absent nodal involvement (*i.e.* T stage > pT2 and pN0) develop distant metastases^6^. Thus, biological markers able to predict prognosis in this subset are still needed.

Prostate-specific membrane antigen (PSMA) is a non-soluble type 2 integral membrane protein with carboxypeptidase activity, expressed on the apical surface of endothelial cells. The expression of this antigen is not fully prostate-specific as it is also expressed in tumor-associated neovasculature of several solid malignancies^7–9^. It is known that PSMA plays a functional role in prostate cancer progression^10^, but its role in BCa progression is not well established yet.

PSMA has been validated as a target for PET imaging of prostate cancer, at primary staging and biochemical recurrence^11,12^. For human the most frequent used PET imaging PSMA ligand is the low-weight urea-based PSMA inhibitor Glu-NH-CO–NH-Lys(Ahx)-HBED-CC labelled with ^68^Gallium (^68^ Ga-PSMA-11). Like the PSMA monoclonal antibody used for immunostaining in our study (*i.e.* SP29), ^68 ^Ga-PSMA-11 binds to a C-terminal epitope of the large extra-membrane domain of PSMA. How much the degree of uptake of ^68 ^Ga-PSMA-11 uptake in BCa significantly correlates with PSMA expression in the different BCa lesions and stage of the disease has to be demonstrated yet.

The aim of the present study was to assess whether PSMA expression could represent a useful marker for BCa diagnosis and to establish whether its expression could be an apt target for anti- angiogenesis and/or radionuclide treatment in BCa.

## Results

The analysis of PSMA expression was feasible in the overall series of tumor and non tumor samples (healthy tissues, hyperplasia, dysplasia). We observed PSMA expression only in the tumor neovasculature; in particular PSMA positivity in the tumors was observed in the tumor neovasculature, while in the non tumor tissue it was expressed in the neovasculature of dysplastic area and in some cases it was observed in ulcerated area. Eighteen samples (20.7%) were classified as non tumor samples of whom 11 (12.6%) were healthy bladder tissue, 3 (3.4%) were hyperplasia and 4 (4.6%) were dysplastic tissue. Sixty-nine (79.3%) were tumor samples (Table 1).

**Table 1:** PSMA expression (H-score*) in neovasculature of bladder tissue samples.

	N. (%)	Median (range)		N. (%)	Median (range)	*P*
Overall samples	87	210 (0–300)	Non tumor samples	18 (20.7)	20 (0–270)	**0.00288**
Healthy tissue	11 (12.6)	0 (0–140)				
Hyperplasia	3 (3.4)	20 (0–300)	Tumor samples	69 (79.3)	225 (0–300)	
Displasia	4 (4.6)	270 (240–270)				

	N. (%)	Median (range)	*P*			
*Tumor type*						
Papillary tumor	39 (62.9)	270 (0–300)	0.0735			
Infiltrating tumor	23 (37.1)	140 (0–300)				
*Grade*						
Low Grade	32 (51.6)	270 (0–300)	0.177			
High Grade	30 (48.4)	180 (0–300)				

	N. (%)	Median (range)		N. (%)	Median (range)	*P*
*Tumor stage*						
Tis	7 (10.1)	200 (0–300)	Low stage	46 (66.7)	232.5 (0–300)	0.719
Ta	15 (21.7)	270 (0–300)				
T1	24 (34.8)	210 (0–300)				
T2	11 (15.9)	200 (0–300)	High stage	23 (33.3)	200 (0–300)	
T3	11 (15.9)	240 (0–300)				
T4	1 (1.4)	140				

*H-score: the product of the percentage of the immunopositive tumor cells and the staining intensity.

Tis: in situ.

No. 69 total tumors: 62 “tumors classified by tumor-type/grade” and 7 “in-situ tumors”.

Out of the 69 tumor samples 7 were in situ carcinomas, 39 were papillary carcinomas of whom 5 had infiltrating morphological features, and 23 were infiltrating tumors. These 23 samples were further classified by the pathologist in: 1 adenocarcinoma of the bladder, 2 small cell carcinomas, 2 squamous cell carcinomas, 1 clear cell urothelial carcinoma (glycogen rich), 1 sarcomatoid carcinoma, 2 poorly differentiated urothelial carcinomas, 1 urothelial carcinoma with glandular differentiation, 10 conventional urothelial carcinomas and 3 large nested urothelial carcinomas.

**Figure 1:**
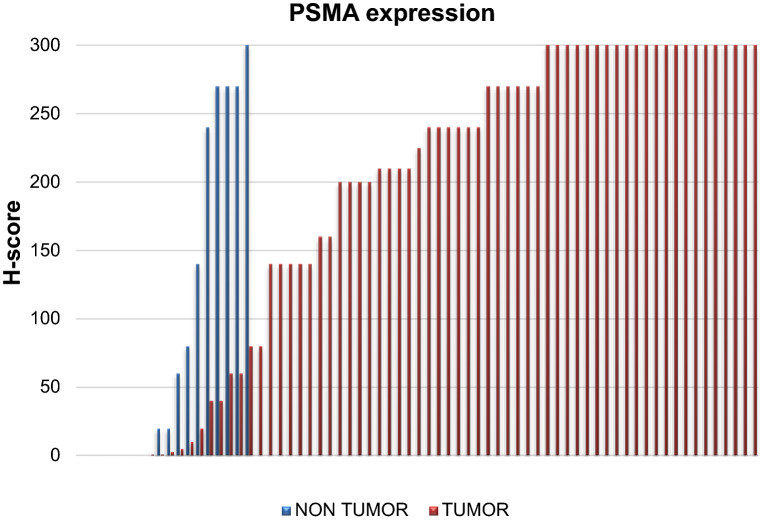
Data distribution in terms of PSMA H-score of non tumor (no. 18) and tumor samples (no. 69).

The median PSMA expression in terms of H-score in the overall series was 210 (range 0–300) (Table 1). In Fig. 1, we present the data distribution in terms of PSMA H-score of non tumor (no. 18) and tumor samples (no. 69).

The median PSMA expression in terms of H-score significantly differed between non tumor and tumor samples (*p* = 0.00288) (Table 1), that showed higher PSMA expression in the tumor neovasculature. We observed higher expression of PSMA in papillary tumors than in urothelial infiltrating tumors, although the difference was not statistically significant (Table 1). No differences were observed for PSMA expression in relation to grade and stage.

**Figure 2:**
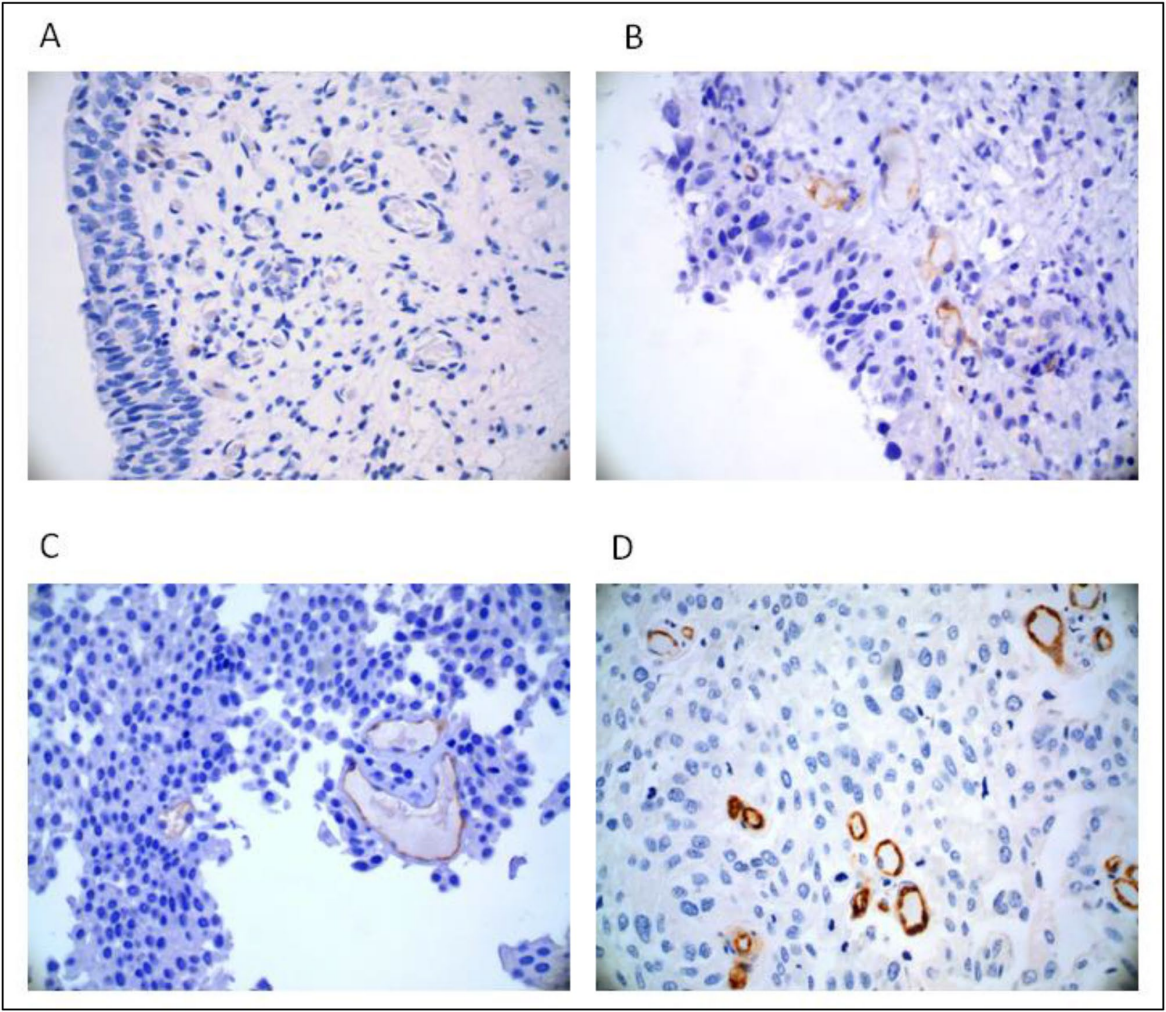
Representative images of PSMA immunohistochemical stainings (40X magnification). (**A**) Chronic erosive cystitis negative for PSMA expression; (**B**) weak PSMA expression of transitional cells in a in situ lesion; (**C**) low grade papillary carcinoma showing a weak PSMA expression; (**D**) strong immunoreactivity of a high grade papillary carcinoma.

**Figure 3:**
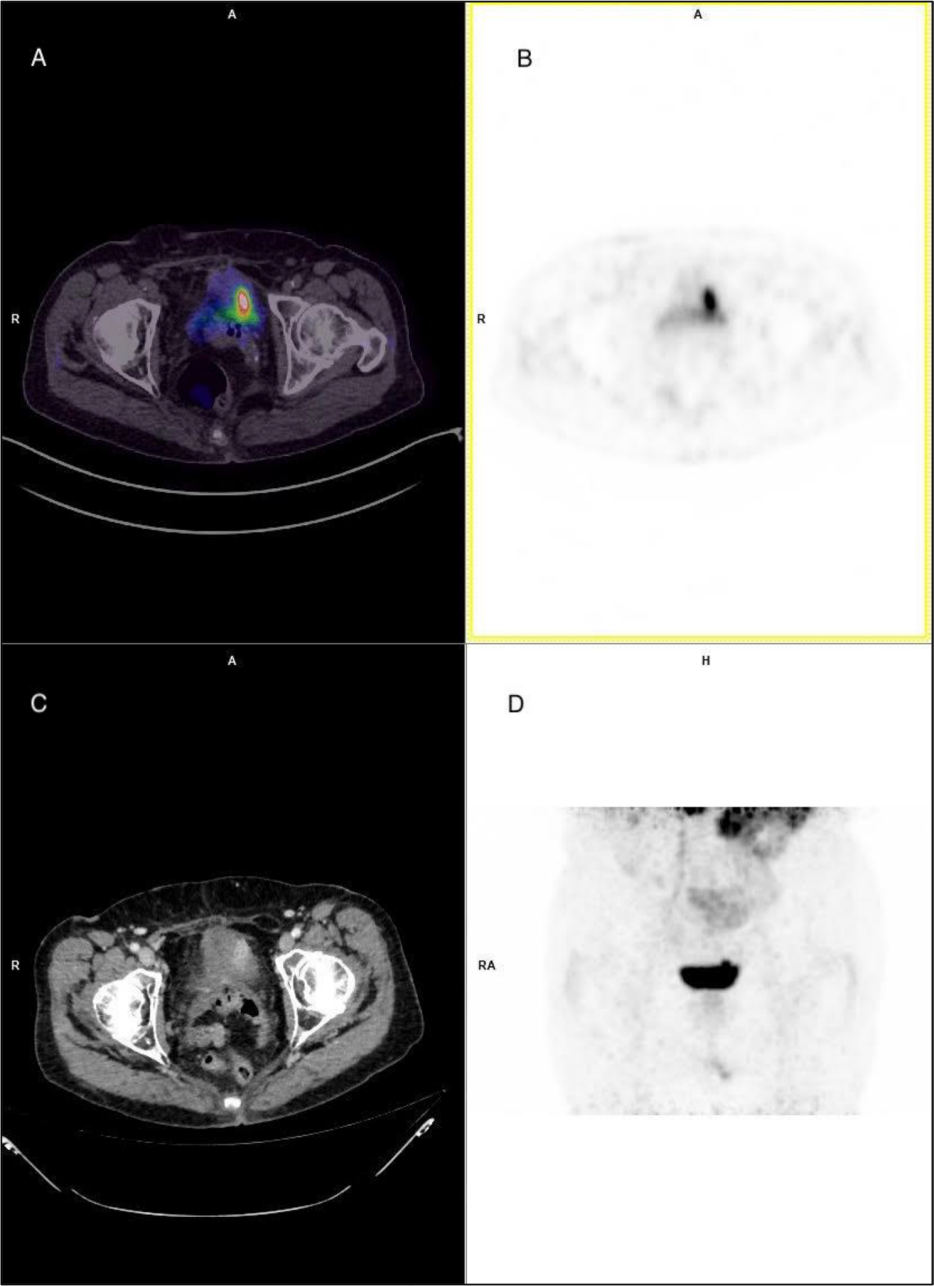
78-year-old patient with high-grade urothelial carcinoma. (**A**) and (**B**) Transaxial ^68 ^Ga-PSMA-11 PET/CT fused and ^68 ^Ga-PSMA-11 PET-only images showing focal high PSMA uptake at the site of the primary BCa (left bladder wall); (**C**) Transaxial contrast-enhanced CT image showing primary BCa enhancing lesion; (**D**) Maximum Intensity Projection (MIP) image of ^68 ^Ga-PSMA-11 distribution in the pelvic area.

In Fig. 2, we reported PSMA expression of a benign lesion and in different tumor samples at different stages, where only neovasculature-related PSMA expression was demonstrated (Fig. 2A–D). Figure 2D shows PSMA expression with a strong immunoreactivity of a high grade solid papillary carcinoma from a 78-year-old patient with high-grade urothelial carcinoma. Figure 3 reports the ^68 ^Ga-PSMA-11 PET/CT of this patient.

## Discussion

The clinical management of patients with BCa is normally based on cytology and cystoscopy. These techniques are not 100% accurate in the diagnosis, localization and discriminating between low- and high-risk patients, indicating the need for companion biomarkers to be used in BCa clinical practice, even more they could serve as therapeutic target. Several molecular tests have been proposed and among them Fluorescence in situ hybridization (UroVysion, Abbott, Vysis) obtained FDA approval for the diagnosis and monitoring of BCa patients^13^. However, its use requires specialized personnel for the analysis and appropriate laboratory equipment. For these reasons, it is not feasible in all the laboratories and the search of new biomarkers is still ongoing.

Despite in vitro and in vivo studies have demonstrated PSMA expression in various malignant and benign tumors as promising biomarker, its role in BCa diagnosis and evolution has yet to be confirmed^10,14,15^. In fact, several studies reported that differences in PSMA expression in terms of cellular or tissue localization may depend on the type of antibody and platform used and sample analyzed. It has also been reported that other healthy and benign tissues different from prostate cancers display PSMA apical and cytoplasmic positivity, and PSMA expression is also observed in the neovasculature^9,16,17^. In the bladder, we demonstrated that PSMA is expressed exclusively in the neoformed vessels of carcinoma, even in in situ tumors, and it showed a weak expression in the vasculature of inflammatory/erosive benign lesions (chronic and erosive cystitis). These results are in accordance with literature data showing that PSMA is a marker of tumor associated neo-angiogenesis. These data could be useful in BCa diagnosis and could acquire even more importance in considering new therapeutic strategies, such as anti-angiogenesis agents and radio-labelled PSMA ligands.

Despite no differences have been observed on PSMA expression exclusively found in tumor neovasculature, in relation to tumor type, grade and stage, we found that PSMA expression, in terms of H-score was different between non tumor samples and tumor samples, reflecting the presence of a neoplasm. In addition, the high PSMA expression observed in the papillary tumors is in accordance with the fact that they are frequently accompanied by abundant fibrovascular supporting stroma. Moreover, the exuberant PSMA-positive neovascularization could be explained by the role of the gene FGFR3 and its mutations at the level of BCa cells, an indirect marker of tumor aggression implicated in the neo-angiogenesis process^18,19^.

We are aware of the retrospective nature of the study and of the limited number of analyzed cases, but this explorative work could thus form the basis for future research to assess the real diagnostic and prognostic value of PSMA on enlarged case series of samples of BCa.

Based on an immunohistochemical study showing PSMA expression in urothelial BCa, some authors hypothesized that PSMA expression in adenocarcinoma of the urinary bladder can be demonstrated in vivo using ^68 ^Ga-PSMA-11PET/CT^19^. Both 18F-FDG and ^68 ^Ga-PSMA-11 PET/CT were performed in a single patient, which showed PSMA expression both at the site of the primary BCa and in metastatic lymph nodes^20^. Similarly, in a 78-year-old patient with high-grade urothelial carcinoma (Fig. 3) we could observe high ^68 ^Ga-PSMA-11 primary tumor uptake by PET, corresponding to high PSMA tumor expression on immunohistochemistry (Fig. 2D). These anecdotical experiences may suggest a role for ^68 ^Ga-PSMA-11 PET/CT for in vivo detection of primary and metastatic BCa. Still, the high renal excretion and bladder accumulation of all ^68 ^Ga-labeled PSMA ligands of clinical use greatly hamper primary urothelial detection due to the very low tumor neovasculature PSMA uptake compared to the considerable urinary PSMA activity background. The increasing, but still limited availability of ^18^F-labeled PSMA ligands with negligible urinary excretion such as ^18^F-PSMA-1007 and, more recently ^18^F-AlF-PSMA-11, provide better accuracy in the visualization of bladder wall lesions along with metastatic disease, where present. Thus, the in vivo confirmation of tumor neo-angiogenesis through ^18^F-PSMA-1007 PET could allow for more accurate pre-operative staging and lesion characterization.

In the clinical scenario of metastatic BCa patients, the verification of PSMA-expressing BCa lesions by either ^68 ^Ga-PSMA or ^18^F-PSMA PET imaging would allow for the evaluation of eligibility for anti-angiogenic agents and PSMA radionuclide therapy. In this specific context, ^68 ^Ga-PSMA-11 urinary excretion would not impede visualization of PSMA-avid metastatic disease. About PSMA radionuclide therapy, established PSMA theranostic tandems such as PSMA I&T and PSMA-617 (labeled with ^68 ^Ga for diagnostic imaging and ^177^Lu for delivering targeted radiation therapy) could be investigated in BCa with future studies both as an alternative to maintenance endovesical treatments of superficial BCa and as a supplementary therapeutic option for metastatic BCa patients. We demonstrated by immunohistochemistry the presence of PSMA in the neovasculature of BCa and demonstrated positive targeting of PET-labeled PSMA ligand. In future the evaluation of tumor-retention time is needed to assess the appropriateness of long half-life therapeutic PSMA ligands as radionuclide treatment for BCa.

## Methods

### Case series

This retrospective study included BCa patients followed up at the Istituto Scientifico Romagnolo per lo Studio e la Cura dei Tumori (Italy) between 2009 and 2020.

Seventy-four patients were enrolled in the study and 87 BCa samples were analyzed. The study protocol was reviewed and approved by the Area Vasta Romagna Ethics Committee. Patients’ median age was 73 years (range 40–93). All diagnostic procedures and pathology analyses were carried out in accordance with the relevant guidelines and regulations, and written informed consent was obtained from all study participants. The histology and grading of 87 lesions were established by an expert pathologist at our hospital in accordance with the International Society of Urological Pathology (ISUP) Consensus Conference guidelines 4^21^.

### Immunohistochemistry

Immunostaining for PSMA expression was performed, as already described in our previous paper^10^, using the Ventana Benchmark Ultra staining system (Ventana Medical Systems, Tucson, AZ, USA) with Optiview DAB Detection Kit (Ventana Medical Systems). Tissue sections were incubated for 32 min with ready-to-use anti-PSMA antibody (SP29 Spring Bioscience, Pleasanton, CA, USA). Sections were automatically counterstained with hematoxylin II (Ventana Medical Systems). PCa and breast cancer tissues were used as positive and negative controls, respectively, in all of the experiments. Biomarker expression was quantified as the percentage of immunopositive tumor cells with membrane staining. Non-malignant tissue around the tumor, when present, was also evaluated for PSMA expression. Staining intensity (*i.e.* 0 absent, 1 weak, 2 moderate, 3 strong) was assessed to calculate the H-score, defined as the product of the percentage of the immunopositive tumor cells and the staining intensity. All samples were evaluated by 2 independent observers. Disagreement of > 10% positive cells was resolved by consensus after joint review using a multihead microscope.

### Statistical analysis

Descriptive statistics are reported as median values and ranges. The normality of data distribution was assessed by Shapiro Wilk test. Since the data were not distributed in a normal way, the relationship between median PSMA expression values and pathology-based categories was evaluated by Mann–Whitney nonparametric test. All *p*-values were based on two-sided testing and values lower than 0.05 were considered statistically significant. Statistical analyses were performed using SAS statistical software version 14 (SAS Inc., Cary, NC, United States of America).
